# Supplementary material for: Crawling and Gliding: A Computational Model for Shape-Driven Cell Migration
Source: PLoS Comput Biol. 2015 Oct 21;11(10):e1004280. doi: 10.1371/journal.pcbi.1004280 (PMC4619082; doi:10.1371/journal.pcbi.1004280)
Supplement: S1 Code — (ZIP) [file pcbi.1004280.s012.zip › release/tst/doc/html/files.html]

Tissue Simulation Toolkit: File List


Tissue Simulation Toolkit
 0.1.4.1


- Main Page
- Namespaces
- Classes
- Files

- File List
- File Members

File List

Here is a list of all files with brief descriptions:

|  |  |
| --- | --- |
| ca.cpp |  |
| ca.h |  |
| cell.cpp |  |
| cell.h |  |
| conrec.cpp |  |
| conrec.h |  |
| crash.cpp |  |
| crash.h |  |
| dish.cpp |  |
| dish.h |  |
| engulfment.cpp |  |
| graph.h |  |
| hull.cpp |  |
| hull.h |  |
| info.cpp |  |
| info.h |  |
| mainpage.h |  |
| misc.cpp |  |
| misc.h |  |
| output.cpp |  |
| output.h |  |
| parameter.cpp |  |
| parameter.h |  |
| parse.cpp |  |
| parse.h |  |
| pde.cpp |  |
| pde.h |  |
| pushing.cpp |  |
| qt3graph.cpp |  |
| qtgraph.cpp |  |
| qtgraph.h |  |
| random.cpp |  |
| random.h |  |
| sorting.cpp |  |
| sqr.h |  |
| sticky.h |  |
| tumor.cpp |  |
| vessel.cpp |  |
| warning.cpp |  |
| warning.h |  |
| x11graph.cpp |  |
| x11graph.h |  |


---

Generated on Thu Aug 14 2014 22:04:02 for Tissue Simulation Toolkit by  

 1.8.6
